# Detection of a novel avian influenza A (H7N9) virus in humans by multiplex one-step real-time RT-PCR assay

**DOI:** 10.1186/1471-2334-14-541

**Published:** 2014-10-08

**Authors:** Jian Fan, David Cui, Siuying Lau, Guoliang Xie, Xichao Guo, Shufa Zheng, Xiaofeng Huang, Shigui Yang, Xianzhi Yang, Zhaoxia Huo, Fei Yu, Jianzhou Lou, Li Tian, Xuefen Li, Yuejiao Dong, Qiaoyun Zhu, Yu Chen

**Affiliations:** Department of Clinical Laboratory, the First Affiliated Hospital, School of Medicine, Zhejiang University, 79 Qingchun Road, Hangzhou, 310003 China; State Key Laboratory for Diagnosis and Treatment of Infectious Diseases, the First Affiliated Hospital, School of Medicine, Zhejiang University, 79 Qingchun Road, Hangzhou, China; State Key Laboratory of Emerging Infectious Diseases, Department of Microbiology, The University of Hong Kong, Hong Kong, China

**Keywords:** Avian influenza, H7N9, Detection, Rnase P, Multiplex real-time RT-PCR

## Abstract

**Background:**

A novel avian influenza A (H7N9) virus emerged in eastern China in February 2013. 413 confirmed human cases, including 157 deaths, have been recorded as of July 31, 2014.

**Methods:**

Clinical specimens, including throat swabs, sputum or tracheal aspirates, etc., were obtained from patients exhibiting influenza-like illness (ILIs), especially from those having pneumonia and a history of occupational exposure to poultry and wild birds. RNA was extracted from these samples and a multiplex one-step real-time RT-PCR assay was developed to specifically detect the influenza A virus (FluA). PCR primers targeted the conserved M and Rnase P (RP) genes, as well as the hemagglutinin and neuraminidase genes of the H7N9 virus.

**Results:**

The multiplex assay specifically detected the avian H7N9 virus, and no cross-reaction with other common respiratory pathogens was observed. The detection limit of the assay was approximately 0.05 50% tissue culture infective doses (TCID_50_), or 100 copies per reaction. Positive detection of the H7N9 virus in sputum/tracheal aspirates was higher than in throat swabs during the surveillance of patients with ILIs. Additionally, detection of the matrix (M) and Rnase P genes aided in the determination of the novel avian H7N9 virus and ensured the quality of the clinical samples.

**Conclusions:**

These results demonstrate that the multiplex assay detected the novel avian H7N9 virus with high specificity and sensitivity, which is essential for the early diagnosis and treatment of infected patients.

**Electronic supplementary material:**

The online version of this article (doi:10.1186/1471-2334-14-541) contains supplementary material, which is available to authorized users.

## Background

The influenza A virus (FluA) is a negative-sense, single-stranded RNA virus belonging to the family *Orthomyxoviridae*. FluA is further classified on the basis of two surface glycoproteins, hemagglutinin (H) and neuraminidase (N) [1, 2]. All subtypes of FluA comprise various combinations of the H and N glycoproteins. Sixteen H subtypes (H1-H16) and nine N subtypes (N1-N9) have been characterized in avian species, while the H17N10 subtype is found in bats [3, 4]. Most subtypes of FluA virus such as H5 and H7 subtypes are not pathogenic in poultry, but outbreaks in poultry and wild birds are correlated with the highly virulent FluA virus, which can cause severe economic losses owing to massive numbers of deaths of domestic poultry [4–6].

Human infections are generally associated with the H1, H2, and H3 subtypes, although sporadic cases or outbreaks of avian FluA subtypes H5N1 [3], H7N2 [5], H7N3 [5], H7N7 [6], H10N8 [7], H10N7 [8], and H9N2 [9] have resulted from direct transmissions from domestic poultry and wild birds to humans. Most patients infected with these subtypes exhibit mild symptoms, such as conjunctivitis and acute upper respiratory tract infections associated with fever and sore throat, with or without gastrointestinal symptoms. However, the H5N1 virus exhibits a high mortality rate of over 50%, the H7N7 virus has caused one fatality, and three deaths have resulted from the H10N8 virus [3, 5, 6].

The avian influenza A (H7N9) virus has been found in poultry and birds, although no human infections have been documented, and its pathogenicity was found to be low in previous studies [4]. Since February 2013, a novel, reassortant avian influenza A (H7N9) virus associated with human deaths has emerged in eastern China [10, 11]. Up to February 28, 2014, this novel, highly virulent H7N9 virus for humans has infected over 381 patients who presented with influenza-like illness (ILI) and severe acquired pneumonia, resulting in 118 deaths. It will be a challenge to control the H7N9 outbreak in humans before it spreads further. Currently, sporadic cases, but no evident outbreaks, of this novel virus have been documented in wild birds and poultry, and there is some limited evidence of human-to-human transmission during unprotected exposure [12], and there are no vaccines for humans.

Thus, early detection and isolation of suspected patients are the most effective measures to control and prevent further transmission of the virus. Here, we report a multiplex real-time RT-PCR assay allowing the simultaneous detection and discrimination of universal FluA strains and the novel H7N9 subtype in a single test tube. The results showed that the sensitivity and specificity of this assay was comparable to a protocol recommended by the World Health Organization (WHO).

## Methods

### Primer and probe design

Primers and probes were designed to correspond to conserved regions of the FluA matrix (M) gene segment. The primers/probes for the novel H7N9 strain were designed to detect subtype-specific H and N gene segments, and the Rnase P gene (RP) served as an internal control to monitor the quality of the clinical specimens. These primers/probes were designed according to the Influenza Primer Design Resource and primers/probes recommended by the WHO protocol were listed in Table 1[13].

**Table 1:** **Primers and probes designed and used in this study**

Set	Primers and probes	Sequences (5′ → 3′)	Targeted genes	Location (bp)	GenBank accession no.
Designed and used in this study					
FluA	FluA forward	GGARTGGMTAAAGACAAGACCAATC		129-153	
	FluA reverse	GGCRTTYTGGACAAASCGTCTAC	Matrix protein	227-249	KC885959.1
	FluA probe	ROX- AGTCCTCGCTCACTGGGCACGGT-BHQ2		199-221	
H7	H7 forward	GAGGCRATGCAAAATAGAATACAGAT		1510-1535	
	H7 reverse	CCGAAGCTAAACCARAGTATCACA	Hemagglutinin	1569-1592	KC885956.1
	H7 probe	FAM- ACCCRGTCAAACTAAGCAGCGGYTAYAA-BHQ1		1538-1565	
N9	N9 forward	GCCCTGATAAGCTGGCCACT		472-491	
	N9 reverse	ACTAGTACTTGACCAMCCAATGCA	Neuraminidase	529-552	KC885958.1
	N9 probe	HEX- TCACCRCCCACAGTRTACAAYAGCA-BHQ1		496-520	
RP	RP forward	AGATTTGGACCTGCGAGCG		50-68	
	RP reverse	GAGCGGCTGTCTCCACAAGT	Ribonuclease P	71-93	NM_006413.4
	RP probe	CY5- TTCTGACCTGAAGGCTCTGCGCG-BHQ2		95-114	
Designed in the World Health Organization (WHO) protocol and used in this study					
FluA	InfA Forward	GACCRATCCTGTCACCTCTGAC		146-167	
	InfA Reverse	AGGGC**A**TTYTGGACAAAKCGTCTA	Matrix protein	228-251	
	InfA Probe	FAM-TGCAGTCCTCGCTCACTGGGCACG-BHQ1		201-224	
H7	CNIC-H7F	GGTTTTTTCTTGTATTTTTATATGACTTAG		468-490	
	CNIC-H7R	GG**T**TTTTTCTTGTATTTTTATATGACTTAG	Hemagglutinin	521-550	
	CNIC-H7P	FAM-AGATAATGCTGCATTCCCGCAGATG-BHQ1		495-519	
N9	CNIC-N9F	T**G**GCAATGACACACACTAGTCA**G**T		929-952	
	CNIC-N9R	ATTACCTGGATAAGGGTC**G**TTACACT	Neuraminidase	1010-1035	
	CNIC-N9P	FAM- AGACAATCCCCGACCGAATGACCC -BHQ1		975-998	
Rnase P	Rnase P Forward	AGATTTGGACCTGCGAGCG		50-68	
	Rnase P Reverse	GAGCGGCTGTCTCCACAAGT	Ribonuclease P	71-93	
	Rnase P Probe	FAM-TTCTGACCTGAAGGCTCTGCGCG-BHQ1		95-114	

The 6th base of the FluA reverse primer was changed from “G” to “A”, the 3rd base of the H7 reverse primer was changed from “C” to “T”, and the 2nd and 23rd bases of the N9 forward primer and the 19th base of the N9 reverse primer were changed from “T” to “G”, “T” to “G”, “A” to “G”, with respect to the novel H7N9 sequences, in the WHO-recommended primers.

### Clinical specimens and patients

According to diagnostic criteria of China for the detection of avian influenza A(H7N9) virus highly virulent in humans, patients with acute respiratory infections with influenza-like illness (ILIs), especially those having pneumonia and a history of occupational exposure to poultry and wild birds, were examined by the Infection Department of the First Affiliated Hospital of the College of Medicine of Zhejiang University. Clinical specimens, including throat swabs, nasal aspirates and washes, and sputum specimens, were collected beginning in April 2013. Moreover, clinical specimens were also collected from patients with confirmed H7N9 viral infections. All specimens were stored at −80°C. Specimens were tested for the presence of FluA and the novel H7N9 subtype using a real-time RT-PCR assay, and highly virulent avian influenza A (H7N9) virus and other common respiratory pathogens were cultured in the State Key Laboratory for Diagnosis and Treatment of Infectious Diseases. According to the Declaration of Helsinki (1964), informed consents for participation were obtained from the participants or guardians of the patients, and the Medical Ethical Committee of our hospital approved this study.

### Viral RNA extraction

RNA from viral cultures of three novel human H7N9 viruses, three human novel H1N1 viruses, five seasonal human H1N1 viruses, five seasonal human H3N2 viruses, three human influenza B viruses, 83 specimens from patients without ILI, and 1,011 specimens from patients with ILI was extracted using the RNeasy Mini Kit (Qiagen, Valencia, CA) according to the manufacturer’s protocol.

### Multiplex real-time RT-PCR assay

The multiplex one-step real-time RT-PCR assays were carried out using the One Step PrimeScript® RT-PCR Kit (Perfect Real Time) (Takara, China). In brief, 1.0 μl of 20 μM FluA, H7, N9 and RP primers, 0.5 μl of the corresponding probes. 5 μl of RNA, 25 μl of 2× One Step RT-PCR buffer, 2 μl of Ex Taq HS Polymerase, 2 μl of PrimeScript RT Enzyme Mix and an appropriate volume of Rnase-free distilled water (dH_2_O) were combined in a 50 μl reaction volume. The thermal cycles were performed on an ABI 7500 real-time PCR system (Applied Biosystems, Foster city, CA) under the following conditions: 30 min at 50°C for reverse transcription; 5 min at 95°C, then 40 cycles at 95°C for 15 s and 55°C for 45 s for PCR amplification. The PCR results were determined if the quality controls work. First, the specimen is positive if Ct value is ≤38.0 with appropriate results. Second, the specimen is negative if the Ct value is undetectable. Third, the specimen with a Ct > 38 is suggested to be repeated, the specimen is considered positive if the repeat result is the same as before, conversely the specimen is considered negative if the repeat result is undetectable.

### Viral culture

Madin-Darby canine kidney (MDCK) cells were used to isolate the novel avian influenza A (H7N9) virus using standard methods in a biosafety level three (BSL-3) laboratory. MDCK cells were inoculated with fresh clinical specimens. When the cytopathogenic effect (CPE) of the virus was observed, cells were harvested and tested for the novel H7N9 virus by PCR assay of WHO protocol. The harvested viruses were clarified by low-speed centrifugation (8000 g/min) and used as viral stocks.

### Sensitivity and specificity

Titers of the novel H7N9 viruses were calculated by determining the 50% tissue culture infective dose (TCID_50_). The sensitivity was determined per reaction using a real-time RT-PCR assay of serial 10-fold dilutions of the novel H7N9 viruses, starting with 5 × 10^3^ TCID_50_ per reaction. In addition, serial dilutions of the RNA transcripts, ranging from 1 × 10^6^ to 1 × 10^1^ copies per reaction were also used to assess the sensitivity of the assay. RNA was transcribed *in vitro* using a recombinant pGEM®-Easy plasmid (Promega, Shanghai, China) and the RiboMAXTM Large Scale RNA Production System-SP6 (Promega) according to the manufacturer’s protocol, as described in previous reports [1, 2]. Moreover, the detection limits of the multiplex real-time RT-PCR assay were directly compared to a widely used real-time RT-PCR assay protocol that is recommended by the WHO.

The specificity of the assay was assessed for the novel avian influenza A H7N9 viruses and 21 other respiratory pathogens and commensal organisms: human seasonal FluA (H1N1 and H3N2 viruses), pandemic H1N1-2009 influenza A virus, influenza B virus, avian H5N1, avian H9N2, avian H5N3, respiratory syncytial virus (types A and B), human rhinovirus, Mycoplasma pneumoniae, Klebsiella pneumoniae, Acinetobacter baumannii, Pseudomonas aeruginosa, Staphylococcus aureus, Candida albicans, Neisseria meningitides, human parainfluenza virus (types 1 and 2), human adenovirus, and human coronavirus (OC43).

### Clinical sensitivity and specificity

One hundred and thirty throat swabs or sputum specimens were tested at the Center for Clinical Laboratory of the First Affiliated Hospital of the Medical School of Zhejiang University and the State Key Laboratory for Diagnosis and Treatment of Infectious Diseases. These specimens were determined to be positive for FluA and the H7N9 subtype specifically by tissue culture, and each sample was blindly tested and compared using the multiplex real-time RT-PCR and the WHO-recommended real-time RT-PCR assays. Moreover, tissue culture results were used as the true result for sensitivity and specificity calculations.

### Evaluation of the multiplex real-time RT-PCR using clinical specimens

One thousand and eleven clinical respiratory samples, including throat swabs, sputum or lower respiratory tract specimens from patients with acute respiratory infections acquired since April 2013 were tested for FluA and the novel H7N9 viruses using the multiplex real-time RT-PCR at the Center for Clinical Laboratory of the First Affiliated Hospital of the College of Medicine of Zhejiang University. Specimens from patients with confirmed H7N9 infections were tested to determine the persistence and distribution of the H7N9 virus and to evaluate the diagnostic abilities of the multiplex and WHO-recommended real-time RT-PCR assays during hospital stays.

To assess the validity of viral RNA amplification by the multiplex real-time RT-PCR assay, the partially amplified DNA products of samples testing positive by the multiplex real-time RT-PCR assay were selected randomly and cloned into the pMD18T vector (Takara, Japan). Plasmids were transformed into *Escherichia coli* and their sequences were verified by Sangon Biotech Co., Ltd. (Shanghai, China).

### Ethics statement

The protocol was approved by the Ethics Committee of the First Affiliated Hospital, School of Medicine, Zhejiang University.

## Results

### Design of specific primers and probes for the novel influenza A (H7N9) virus

In the WHO protocol, the novel H7N9 virus was detected using a real-time RT-PCR assay that requires four individual tubes, which increases the experimental procedures and expenses. Therefore, using novel H7N9 sequences submitted to the National Center for Biotechnology Information (NCBI) database, we designed a new set of primers and probes to specifically detect the novel H7N9 virus by a multiplex real-time RT-PCR assay in one reaction tube (Table 1). As recommend by the WHO protocol, the primers and probes were designed for the universal detection of FluA, and the primers and probes for RP were used as an internal control to evaluate the quality of the clinical specimens. The primers and probes for the H7 and N9 genes were designed within certain regions of the hemagglutinin (HA) and neuraminidase (NA) genes that are relatively conserved among all H7N9 subtypes, thus allowing the detection of all human and avian H7N9 viruses. As expected, viral RNA samples extracted from novel human H7N9 isolates (A/Zhejiang/DTID-ZJU01/2013) were detected by the multiplex assay (data not shown).

### Sensitivity and specificity of multiplex real-time RT-PCR assay for the novel H7N9 virus

In the multiplex real-time RT-PCR assay for detecting novel H7N9 viruses, the sensitivities of the FluA, H7, and N9 primer and probe sets were all 5 × 10^−2^ TCID_50_ per reaction, and the detection limits for the RNA transcripts were all 1 × 10^2^ copies/per reaction (Table 2). Interestingly, the same detection limits were found for FluA, CNIC-H7 and CNIC-N9 assays in a previous study [13, 14]. Similarly, the specificity of the multiplex assay was evaluated individually using H7N9 virus culture and RNA transcripts. With respect to specificity, the multiplex assay detected the novel H7N9 virus, the FluA (H1N1 and H3N2) viruses and the novel influenza A H1N1 (2009) virus, and no cross-reactions were observed with the panel comprising 21 other respiratory pathogens or commensals. The specificity of the multiplex assay was similar to that of the WHO-recommended assay. Thus, these results indicate that the multiplex assay designed in this study is sufficiently sensitive and specific to be used to detect the novel H7N9 virus. Experiments were repeated at least three times.

**Table 2:** **Multiplex PCR for detecting the novel H7N9 virus (TCID**_**50**_ **) and RNA transcripts (copies)**

Name	Copies/per reaction		TCID_50_/per reaction	
	Multiplex	WHO	Multiplex	WHO
Flu A	1 × 10^2^	1 × 10^2^	5 × 10^−2^	5 × 10^−2^
H7	1 × 10^2^	1 × 10^2^	5 × 10^−2^	5 × 10^−2^
N9	1 × 10^2^	1 × 10^2^	5 × 10^−2^	5 × 10^−2^

### Clinical sensitivity and specificity of multiplex real-time RT-PCR assay

Fifteen positive specimens out of 130 clinical specimens were positively identified by the multiplex and WHO-recommended real-time RT-PCR assays; all were FluA, with four novel H7N9 viruses and 11 other FluA subtypes. In addition, these 11 FluA subtypes consisted of three pandemic H1N1-2009 influenza A viruses, seven seasonal H3N2 viruses, and one seasonal H1N1 virus, and were certified by other real-time RT-PCR assays in previous reports [1, 2]. In contrast, tissue culture methods detected only 13 positive FluA viruses out of the 130 clinical specimens. Of the two samples that tested positive with the multiplex assay and negative by tissue culture, one contained one pandemic H1N1-2009 influenza A virus and the other contained a seasonal H3N2 virus; these two indeterminate tissue culture results were subsequently confirmed to be positive by genomic sequence analysis and were excluded from the sensitivity and specificity calculations. Interestingly, four specimens that tested positive for the novel H7N9 virus were uniformly detected by all of the assays. In comparison to the tissue culture method, the multiplex assay and WHO-recommended protocol had the same sensitivity (100%) and specificity (98.3%) for FluA, and the same sensitivities and specificities for the H7 (100%) and N9 (100%) genes in the clinical specimen (Table 3). The cycle threshold (Ct) values, defined as the number of cycles required for the fluorescent signal to cross the detection threshold, of FluA and the H7 and N9 genes, and RP gene of the novel H7N9 virus did not obviously differ between the multiplex assay and WHO-recommended protocol (Table 4). These tests by PCR assay were repeated at least three times.

**Table 3:** **Performance of multiplex PCR in comparison to tissue culture for 130 clinical specimens**

Virus	Culture		Multiplex		WHO		Sens. M/W	Spec. M/W
	+	-	+	-	+	-		
FluA	13^a^	117	15	115	15	115	100/100	98.3/98.3
H7	4	126	4	126	4	126	100/100	100/100
N9	4	126	4	126	4	126	100/100	100/100

Sens., sensitivity; Spec., specificity; M/W, Multiplex/WHO. Values refer individually to the virus culture.

^a^Two negative samples by tissue culture consisted of one H1N1 (2009) and one H3N2 virus by genomic sequence analysis. Indeterminate results were excluded from sensitivity and specificity calculations.

**Table 4:** **Detection of four clinical specimens infected with the novel H7N9 virus by multiplex PCR and WHO assay**

Specimens ID	Multiplex				WHO			
	Flu A	H7	N9	RP	Flu A	H7	N9	RP
20130405-5	24.26 ± 0.35^a^	24.45 ± 0.21	25.03 ± 0.3	20.83 ± 0.3	24.77 ± 0.31	25.07 ± 0.15	24.85 ± 0.5	21.03 ± 0.3
20130406-32	26.43 ± 0.21	26.32 ± 0.31	26.23 ± 0.36	22.48 ± 0.35	26.43 ± 0.26	25.98 ± 0.15	26.52 ± 0.26	22.23 ± 0.15
20130407-69	20.15 ± 0.3	20.37 ± 0.32	20.7 ± 0.4	24.23 ± 0.32	19.78 ± 0.35	20.68 ± 0.36	20.07 ± 0.21	23.66 ± 0.31
20130409-92	31.33 ± 0.26	30.65 ± 0.36	31.23 ± 0.32	26.72 ± 0.4	30.85 ± 0.31	31.04 ± 0.5	31.2 ± 0.32	26.17 ± 0.25

^a^Ct (cycle threshold) mean value ± SD.

### Detecting clinical specimens using multiplex real-time RT-PCR assay

**Figure 1:**
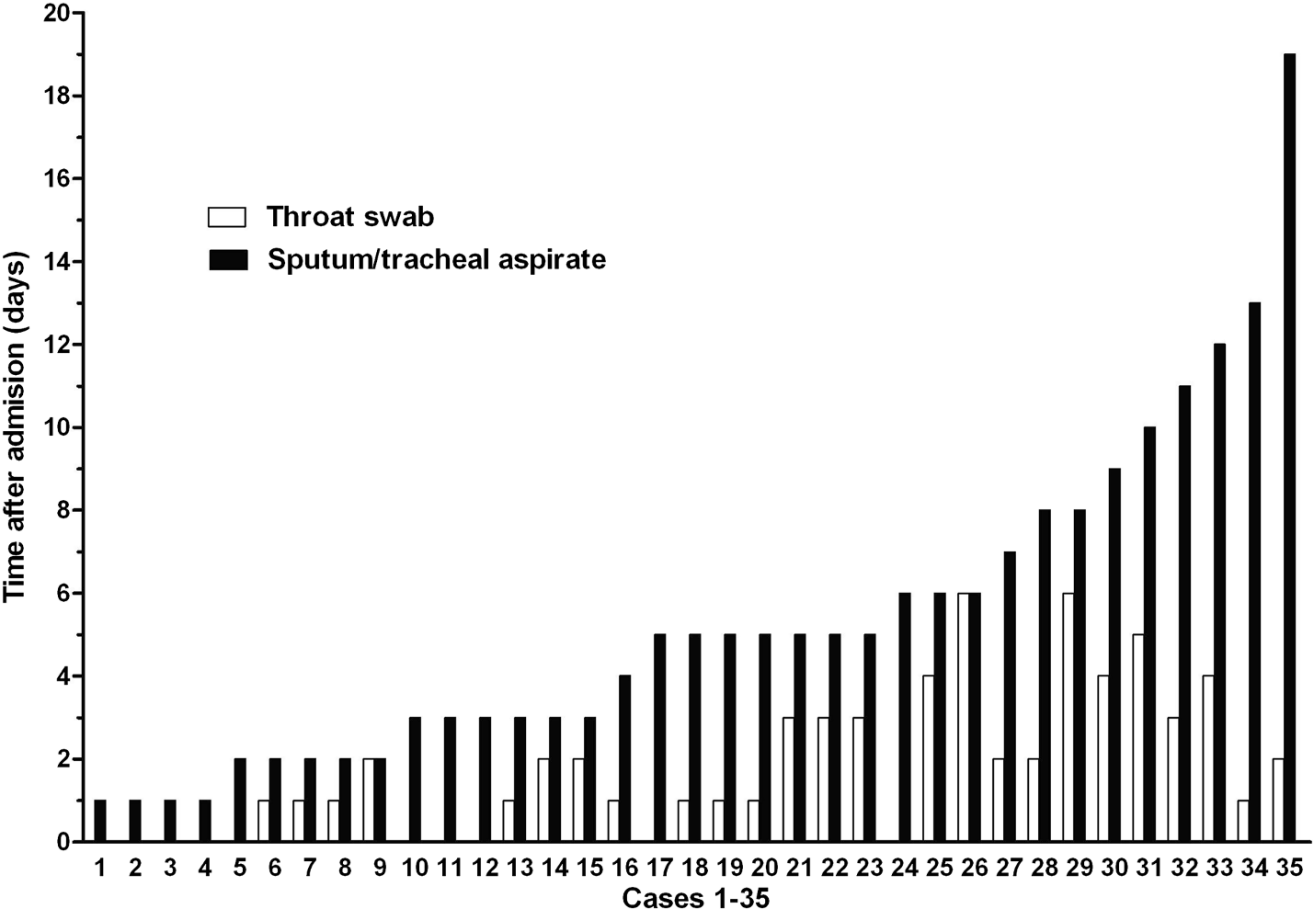
**Surveillance of novel H7N9 viruses from the patients by multiplex real-time RT-PCR.** The average number of days that patients tested positive for H7N9 viruses in sputum or tracheal aspirates specimens (mean value =6.14 days) were significantly greater than that for patients’ throat swabs samples (mean value =2.42 days).

To further demonstrate assay sensitivity and specificity, 1,011 new specimens were tested in parallel using the multiplex real-time RT-PCR and the WHO-recommended real-time RT-PCR protocol. The results showed that, using the multiplex assay, 192 specimens tested positive for FluA, including 35 specimens that were positive for the H7N9 virus. No co-infections with viruses were found in the multiplex tests, 819 samples tested negative for FluA, and the Ct values of RP gene were all positive with 18.5 ~ 29.6 for 1,011 clinical specimens (Table 5). In comparison, the Ct values of RP gene were also all positive with 18.6 ~ 29.8 in 1,011 samples, 192 specimens tested positive for FluA using the WHO protocol. With respect to the H7 gene, both assays identified 35 positive samples. However, the multiplex assay also detected 35 positive samples with respect to the N9 gene, while the WHO protocol only detected 32 positives, and the Ct value of N9 gene in three patients was high with 36 ~ 38 indicating low titres of the H7N9 virus. In an addition, to demonstrate whether the three N9 samples that differed between the two assays were positive or negative, firstly, we amplified DNA products using N9-specific primers, and DNA sequencing of the cloned products revealed that these three samples were confirmed to be positive. Secondly, the sputum of the three cases was individually re-collected and re-detected again, and the Ct values of the three cases in N9 gene were all less than 34. In the three cases, the Ct values of RP gene were individually 27.5 ~ 28.5 (the early samples) and 26.8 ~ 28.5 (the late samples) in the twice samples. Moreover, 35 patients with confirmed novel H7N9 virus infections accepted hospital treatments and their clinical specimens (including throat swab [135 specimens], sputum or tracheal aspirates [160 specimens], etc.) were tested for the presence and distribution of the H7N9 virus by the multiplex and WHO-recommend real-time RT-PCR assays every day following admission, which contributed to the evaluation of therapeutic treatments received by the patients. Eight fecal samples from the 35 patients tested positive for the H7N9 virus using the multiplex assay, while only seven tested positive using the WHO-recommend assay. No positive results for the H7N9 virus were found in blood (35 specimens), urine (30 specimens), cerebrospinal fluid (one specimen), pleural fluid (one specimen) and bone marrow specimens (two specimens). The results of the throat swabs and sputum or tracheal aspirates specimens from 35 patients with H7N9 infections were shown in Figure 1. The results demonstrate that the H7N9 virus persisted in sputum or tracheal aspirate specimens for an average of 6.14 days, while the virus persisted in throat swab samples for 2.42 days.

**Table 5:** **Comparison of the multiplex assay and WHO assay for the detection of H7N9 virus in clinical specimens**

Virus	Multiplex		WHO		Sensitivity (%)	Specificity (%)
	+	-	+	-		
FluA	192^a^	819	192^a^	819	100	100
H7	35	976	35	976	100	100
N9	35	976	32^b^	979	91.4	100
RP	1011	0	1011	0	100	100

^a^Influenza A (FluA) virus positive specimens, including H7N9.

^b^Three specimens with negative N9 and positive H7.

## Discussion

In March 2013, the novel reassortant avian influenza A (H7N9) virus firstly occurred in three persons in Shanghai of China, and then more and more peoples were killed by the novel virus that had been found in ducks, chickens and birds. Until Jul 31, 2014, 413 infections and 157 deaths by the novel H7N9 virus were reported in China. Additionally, several family clusters of avian influenza A (H7N9) virus infection were reported during the outbreak of this virus in China [12]. Most of the cases of novel avian H7N9 infections reported were virulent, and the deaths resulted from the rapid development of severe pneumonia and acute respiratory distress syndrome (ARDS) [11, 12, 15–19].

Tissue culture is the “gold standard” for the diagnosis of viral respiratory infections. However, PCR is significantly more sensitive and highly specific for the detection of respiratory viruses. Therefore, research into PCR techniques for detecting viral infections is conducted in many laboratories [1, 2]. There is an urgent need to develop a rapid and specific molecular test for the early detection and confirmation of the novel avian influenza A (H7N9) virus. In the earlier periods of the outbreaks of this virus, one real-time RT-PCR method, recommended by the WHO, was promptly announced for the detection of novel influenza A (H7N9) viruses [13].

Subsequently, additional real-time RT-PCR methods for the detection of avian influenza A (H7N0) were published as the virus emerged in China [14, 19–24]. These methods were only applied to the analysis of the H7 and N9 genes of the H7N9 virus and did not include the M gene, which aids in the determination of novel H7N9 viruses. Nor did they use the RP gene, which can be used as a control to monitor the quality of clinical samples to ensure accurate results [1, 2]. Additionally, the sequences of the primers recommended by the WHO contained slight discrepancies from those found in publicly available gene databases of novel H7N9 viruses isolated from human cases. Thus, the M, H, and N primer sets in the WHO protocol do not exactly match the sequences of the novel H7N9 virus (Table 1) [13]. For example, the 6th base of the FluA reverse primer was changed from “G” to “A”, the 3rd base of the H7 reverse primer was altered from “C” to “T”, and the 2nd and 23rd bases of the N9 forward primer and the 19th base of the N9 reverse primer were altered from “T” to “G”, “T” to “G”, and “A” to “G”, respectively, relative to the novel H7N9 sequences. These errors may adversely affect viral detection in clinical specimens. Therefore, the nucleotide sequences of primers and probes should be modified according to the variation of novel influenza virus H7N9 sequences.

In our study, a multiplex real-time RT-PCR assay was developed to simultaneously detect universal FluA strains, as well as the novel H7N9 virus, in one reaction tube. The primers and probes in the multiplex assay were designed using the Influenza Primer Design Resource. The H7 and N9 primers and probes exactly match the sequences of the novel high virulent H7N9 virus for humans and low pathogenic avian H7N9 virus, and the FluA primers and probes completely match the M gene of most FluA viruses using base degeneracy rules (Table 1). For example, the 6th base of the H7 forward primer and 15th base of the H7 reverse primer are “R”, indicating “A” or “G”, and the 5th, 23rd and 26th bases of the H7 probe are “R”, “Y” and “Y”, which indicate “A” or “G”, “C” or “T” and “C” or “T”, respectively. These changes contribute to the reliable detection of the virus by a PCR assay.

To confirm its sensitivity and specificity, the multiplex real-time RT-PCR assay was compared to the WHO-recommended assay. The limits of detection of the H7 and FluA genes, with respect to the detection of novel H7N9 viruses and related RNA transcripts, did not significantly differ between the two assays. The Ct values for the amplification of the H7, N9, and FluA genes were generally uniform in the multiplex assay, and were similar to the Ct values of the CNIC H7 and CNIC FluA primers used in the WHO protocol. Additionally, the sensitivity and specificity of the multiplex assay, with respect to FluA, H7, and N9, were equivalent to those of the WHO protocol; no cross-reactions were observed with other viruses and pathogens.

Since March 2013, the National Health and Family Planning Commission of the People’s Republic of China has paid great attention to the increasing number of H7N9 cases and has taken several measures, including specimen testing, clinical treatments, epidemiological investigations, and medical observations of close contacts. The surveillance of pneumonia cases of unknown origin and influenza-like illness with consecutive days of high fever (>38°C) and a history of close contact with poultry has also been strengthened to detect the novel H7N9 virus.

The multiplex real-time RT-PCR assay and the real-time RT-PCR assay recommended by the WHO were used to detect, in parallel, the presence of the H7N9 virus in 1,011 clinical specimens, and 35 patients with the novel H7N9 virus were found. The positive results of the multiplex real-time RT-PCR assay were generally in accordance with that of the real-time RT-PCR assay in the WHO protocol. As previously noted, the Ct values for the amplification of the H7, N9, FluA and RP genes in the two assays were generally similar. In an addition, the Ct values for RP gene assessed by both assays were low with 18.5 to 29.8 in these clinical specimens, which indicated good qualities of clinical specimens. A reliable quality of clinical specimens is important for the determination of the result, and negative result of it has an effect on the assay of objective result in some clinical specimens.

Interestingly, the WHO protocol resulted in three indeterminate results with respect to the detection of the N9 gene. These samples were subsequently considered positive based on genomic DNA sequencing, and samples of the three cases were positive when the sputum was re-collected and re-tested, respectively, so that their mischaracterization was factored into the sensitivity and specificity calculations. We suggest that the use of mismatched primers and probes, as well as extremely low viral concentrations, may have led to this discrepancy. The samples of uncertain results should be re-collected and re-tested, which were significant for the determination of the results.

Different clinical specimens from the 35 hospitalized patients with laboratory-confirmed H7N9 infections were tested with the two assays. The results showed that the multiplex assay and the WHO-recommended assays did not significantly differ in their ability to detect H7N9 viruses; only one positive fecal sample detected by the multiplex assay was negative using the WHO protocol. Remarkably, the percentage of positive sputum or tracheal aspirate specimens (100%) was higher than that of the throat swabs samples (71.43%) on the first day after hospital admission, and the average number of days that patients’ sputum or tracheal aspirate samples tested positive for H7N9 viruses (mean value =6.14 days) was significantly greater than that of the patients’ throat swabs samples (mean value =2.42 days). This may result from the ability of the novel reassortant H7N9 virus to easily infect human lung tissue, which leads to severe pneumonia [10, 11, 15–18].

These results indicate that the multiplex assay designed in this study is sufficiently sensitive and specific to be used for the detection of the novel H7N9 virus. Moreover, in comparison with a single real-time RT-PCR assay, the merits of the multiplex real-time RT-PCR assay applied in our study include the rapid detection and discrimination of human FluA, and novel avian H7N9 viruses using H7 and N9 gene-specific primers in a single tube, which significantly simplifies the experimental procedures and notably reduces experimental expenses.

## Conclusion

In summary, a sensitive and specific multiplex real-time RT-PCR assay was developed for the early detection of the novel reassortant H7N9 virus in patients. This assay will enable the timely evaluation of antiviral pharmaceutical therapies and other therapeutic interventions in the near future. The multiplex RT-PCR assay will be of great importance for public health officials during viral outbreaks.

## Authors’ original submitted files for images

Below are the links to the authors’ original submitted files for images. Authors’ original file for figure 1 Authors’ original file for figure 2
