# Supplementary figures and images for: Detection of a novel avian influenza A (H7N9) virus in humans by multiplex one-step real-time RT-PCR assay
Source: BMC Infect Dis. 2014 Oct 8;14:541. doi: 10.1186/1471-2334-14-541 (PMC4286936; doi:10.1186/1471-2334-14-541)

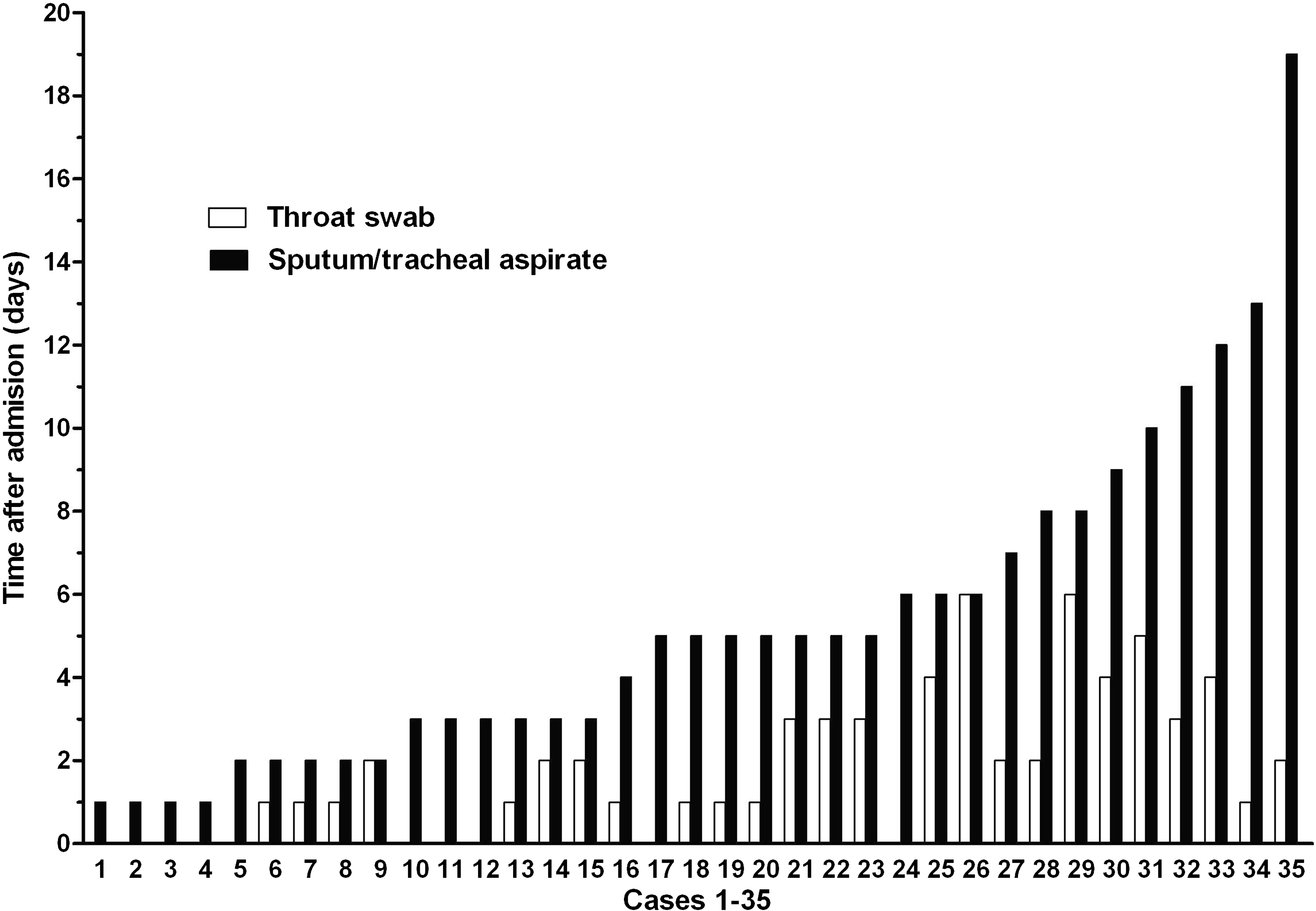

Supplement: Supplementary file 1 — Authors’ original file for figure 1 [file 12879_2014_3856_MOESM1_ESM.tif]

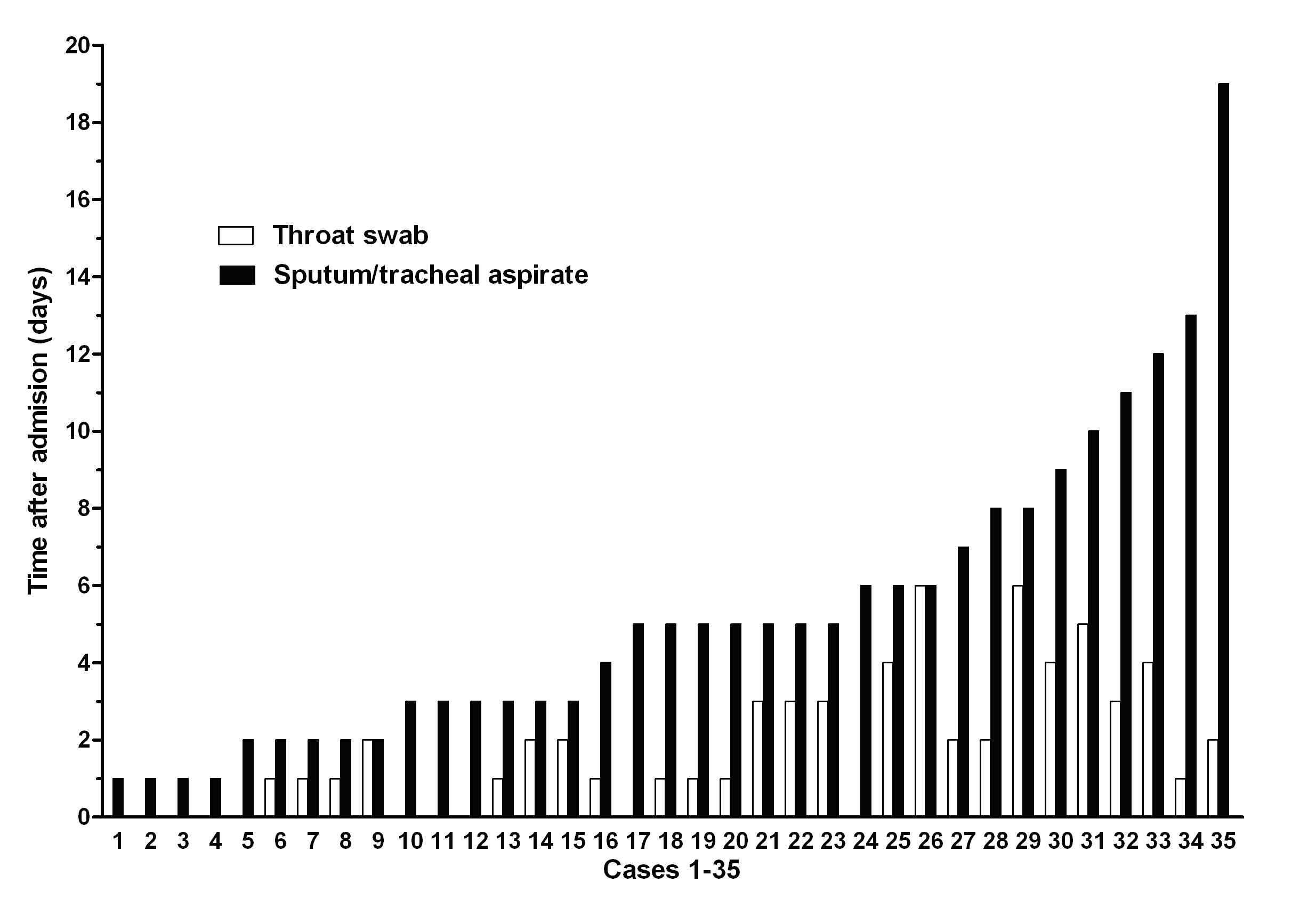

Supplement: Supplementary file 2 — Authors’ original file for figure 2 [file 12879_2014_3856_MOESM2_ESM.jpeg]
